# TFCP2 Fusion-Positive Rhabdomyosarcomas: A Report of 10 Cases and a Review of the Literature

**DOI:** 10.3390/cancers17091441

**Published:** 2025-04-25

**Authors:** Madison P. Ginn, Ryan A. Denu, Davis R. Ingram, Khalida M. Wani, Alexander J. Lazar, Douglas J. Harrison, Michael S. Nakazawa, Anthony P. Conley, Shreyaskumar Patel, John Andrew Livingston

**Affiliations:** 1 Departments of Internal Medicine & Pediatrics, McGovern Medical School, The University of Texas Health Science Center at Houston, Houston, TX 77030, USA; madison.p.ginn@uth.tmc.edu; 2 Division of Cancer Medicine, The University of Texas MD Anderson Cancer Center, Houston, TX 77054, USA; 3 Department of Pathology, Division of Pathology & Laboratory Medicine, The University of Texas MD Anderson Cancer Center, Houston, TX 77030, USA; 4 Department of Genomic Medicine, Division of Cancer Medicine, The University of Texas MD Anderson Cancer Center, Houston, TX 77030, USA; 5 Department of Pediatrics Patient Care, Division of Pediatrics, The University of Texas MD Anderson Cancer Center, Houston, TX 77030, USA; 6 Department of Sarcoma Medical Oncology, The University of Texas MD Anderson Cancer Center, Houston, TX 77030, USA

**Keywords:** TFCP2, fusion, rhabdomyosarcoma, sarcoma

## Abstract

**Background/Objectives:** The fusion of the *TFCP2* gene with either *EWSR1* or *FUS* typically results in a spindle cell and/or epithelioid variant of rhabdomyosarcoma. This is an ultra-rare type of sarcoma, with most of our knowledge about these coming from case reports and small case series. Herein, we describe the clinical characteristics and treatment course of 10 patients with *TFCP2* fusion sarcomas. **Methods**: We identified 10 patients in our hospital system with *TFCP2* fusion sarcomas and 43 previously reported cases in the literature. We assessed primary tumor characteristics, treatment regimens, and survival rates among all cases. **Results:** We find that *TFCP2* fusion sarcomas most commonly occur in young adults (median age: 33 years) and arise in craniofacial bones (7/10, 70%). Concomitant *ALK* alterations and ALK overexpression is nearly universal, and two of our patients were treated with ALK inhibitors; one patient had a near complete response before eventual progression, while the other patient had progressive disease after 2 months. For most, the prognosis was poor. The median overall survival in this cohort was 24.7 months (range: 5.9–29.7 months). Four patients were treated with upfront surgery, and all four developed recurrent disease. The median time to recurrence following upfront surgery was 2.1 months (range: 0.73–6.9 months). Five patients received systemic therapy, and the median progression-free survival from the start of treatment to progression was 1.6 months (range: 0.97–2.7). We also review the 53 total cases of *TFCP2* fusion sarcomas in the literature, again highlighting the dismal outcomes in this disease. **Conclusions:** *TFCP2* fusion sarcomas are proven to be aggressive and have poor prognosis. Additional work is needed to define the optimal treatment course for *TFCP2* fusion sarcomas.

## Simple Summary

Rhabdomyosarcomas with *TFCP2* fusions comprise an extremely rare and aggressive type of cancer. In this paper, we detail the history and treatment of 10 patients with this rare sarcoma and compare them to 53 total reported cases in the literature. *TFCP2* fusion sarcomas are associated with poor outcomes, high rates of recurrence, and treatment resistance. The median overall survival of our cohort was 24.7 months, the median time to recurrence following surgery was 2.1 months, and the median time to progression during treatment with chemotherapy was 1.6 months. As ALK alterations commonly co-occur in these sarcomas, two of our patients were treated with ALK inhibitors, with initial, although short-lived, benefits. More work must be conducted to determine the best treatment course for this disease.

## 1. Introduction

*TFCP2* (aliases LSF and SEF) was first described as an activator of the late SV40 promoter and later found to bind globin genes and HIV-1 promoters [1,2]. *TFCP2* is part of the TFCP2/Grainyhead transcription factor family. It has been implicated as an oncogene in hepatocellular carcinoma [3], pancreatic ductal adenocarcinoma [4], and breast cancer [5], although it has been hypothesized to be a tumor suppressor in melanoma [6]. *TFCP2* is ubiquitously expressed and involved in cell cycle regulation and the expression of lineage-specific genes [7].

The translocation of *TFCP2* (usually exon 2) with either FET family member—*EWSR1* (usually exon 5) or *FUS* (usually exon 6)—most often results in an epithelioid variant of rhabdomyosarcoma. The resulting fusion protein retains the CP2 DNA binding and the SAM/pointed domains of *TFCP2* [8]. Pathologically, they are notable for significant ALK overexpression, as well as often expressing desmin, myogenin, MyoD1, and cytokeratin [9]. This is a rare orphan disease, so most of what is known comes from case reports and small case series. *TFCP2*-fusion sarcomas most commonly occur in adolescent and young adult women, although one case was reported in a 72-year-old person [10]. They most commonly arise in a craniofacial bone, such as the mandible, but have also been reported in the pelvis and chest wall [11,12,13,14]. *TFCP2* fusion sarcomas are associated with extremely poor prognosis [14]. Patients who present with localized disease harbor a high risk of development of distant metastases (over 50%) [15]. The median overall survival has been reported to be 29 months [15]. Furthermore, responses are rare to traditional combination chemotherapy utilized for other rhabdomyosarcomas, and median progression-free survival on these treatments is about 2 months [16].

Herein, we report the clinical characteristics, treatments, and outcomes of 10 patients with *TFCP2* fusion sarcomas from a single institution.

## 2. Methods

### 2.1. Study Design

This is a retrospective study of patients seen at MD Anderson Cancer Center from 2016–2024 with a *TFCP2* fusion sarcoma. Electronic medical records from the identified patients were reviewed manually. The following clinical data were collected via retrospective chart review: age, gender, race, ethnicity, date of birth, date of diagnosis, vital status, tumor grade, tumor mitotic rate, tumor stage, surgery date, systemic therapies and dates of treatment, response to treatment, imaging results, personal history of cancer, family history of cancer, and results of next-generation sequencing tests. The response to treatment was determined based on the treating clinician’s determination and description in the electronic medical record.

### 2.2. Ethics

This study was approved by the University of Texas MD Anderson Cancer Center (MDACC) Institutional Review Board (protocol 2022-0278, approved 9 December 2022, and protocol DR09-0245, approved 26 May 2009) and was conducted in accordance with the U.S. Common Rule. Clinical and genomic data were obtained following signed informed consent onto prospective institutional protocols or under retrospective review protocols with a limited waiver of authorization.

### 2.3. Data Analysis and Statistics

Statistical analyses were performed using GraphPad Prism (version 9.5.0 or higher, RRID:SCR_002798) and R (version 4.2.2 or higher). The ggplot2 package was used to construct a swimmer plot.

Overall survival (OS) was calculated from the date of the first histologic diagnosis (either pre-treatment biopsy or surgical pathology) to the date of death or the latest follow-up (whichever occurred earlier). Recurrence-free survival (RFS) was calculated in patients with initially localized disease from the date of surgery for resection of the primary tumor to the date of recurrence or the latest follow-up (whichever occurred earlier). Progression-free survival (PFS) was defined in patients with metastatic disease from the start of therapy to the date of progression or the latest follow-up (whichever occurred earlier).

### 2.4. Data Availability

Deidentified data leading to the reported findings in this paper are available upon request from the corresponding author.

## 3. Results

### 3.1. Clinico-Pathologic Features of TFCP2 Fusion-Positive Sarcomas

**Figure 1:**
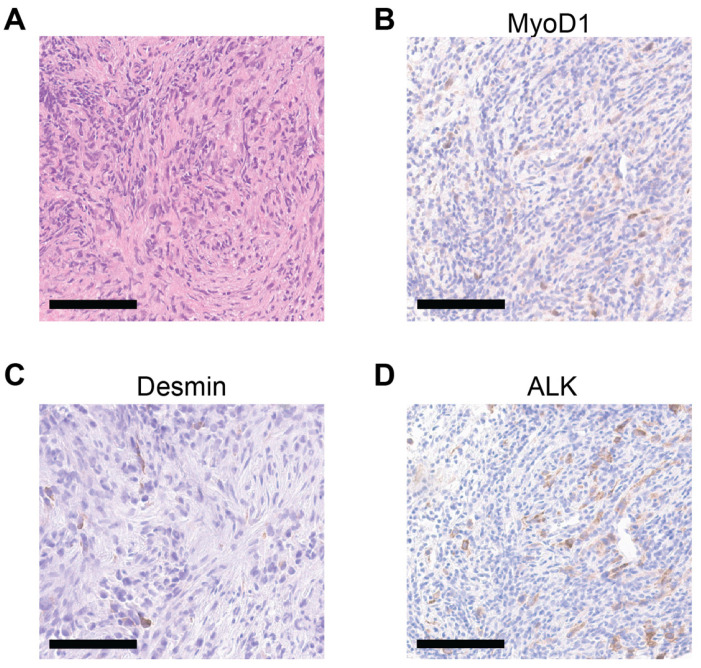
Pathologic features of *TFCP2* fusion sarcomas. (**A**) Representative H&E image of a *TFCP2* fusion-positive spindle cell/sclerosing rhabdomyosarcoma. (**B**–**D**) Representative images of immunohistochemical staining for MyoD1 (**B**), desmin (**C**), and ALK (**D**). Scale bars = 100 μm.

**Figure 2:**
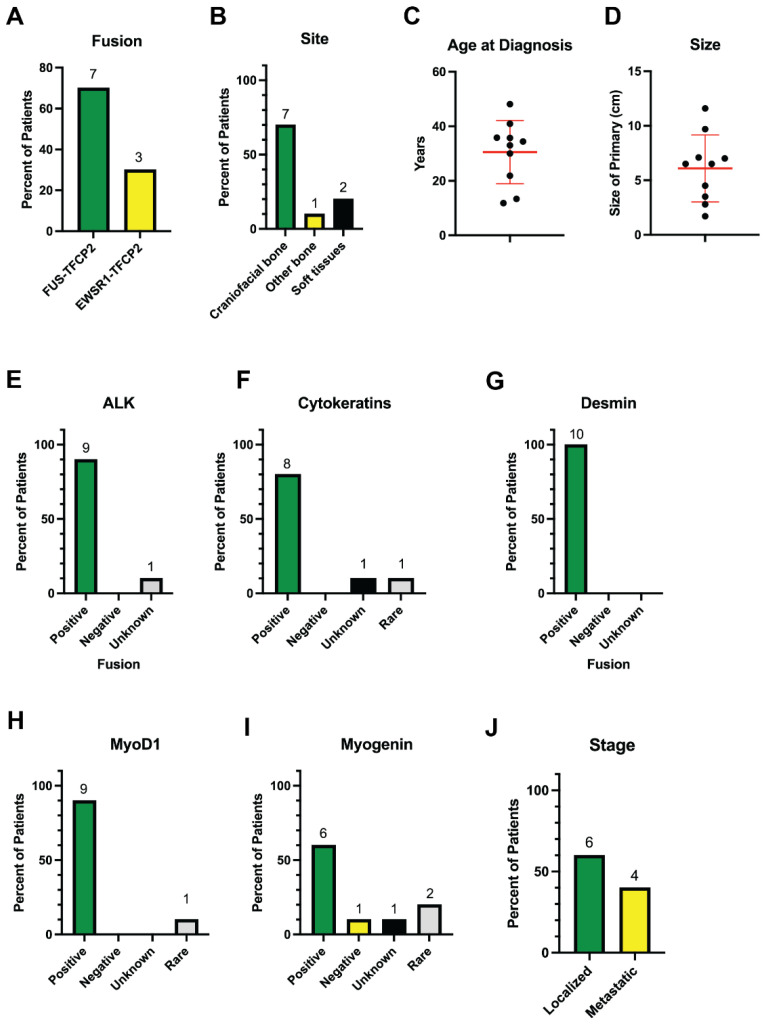
Clinical and pathologic characteristics of *TFCP2* fusion sarcomas. (**A**) Percent of patients with each of the two *TFCP2* fusions, either *FUS-TFCP2* or *EWSR1-TFCP2*. (**B**) Breakdown of the site of disease. (**C**) Distribution of the age at diagnosis. (**D**) Distribution of the size of the sarcoma at diagnosis. In (**C**,**D**), bars represent means ± standard deviations, and each black dot represents a different patient. (**E**–**I**) Percent of tumors that stained positive for ALK (**E**), cytokeratins (**F**), desmin (**G**), MyoD1 (**H**), and myogenin (**I**). (**J**) Disease stage at diagnosis. The numbers on top of the bars indicate the absolute number of patients represented by the bar.

We identified 10 patients with confirmed *TFCP2* fusion-positive sarcomas (Table 1). Most exhibited a fascicular spindle cell morphology (Figure 1A) and stained positive for MyoD1 (Figure 1B), desmin (Figure 1C), and ALK (Figure 1D). Seven had the *FUS-TFCP2* fusion, and three had the *EWSR1-TFCP2* fusion (Figure 2A). Regarding sites of disease, seven (70%) arose in craniofacial bones, one (10%) started in the acetabulum, and two (20%) started in soft tissues of the trunk (Figure 2B). The mean and median ages of diagnosis were 30.5 and 33.0 years, respectively (range: 11.8–48.1 years; Figure 2C). The mean and median sizes of the primary tumor at diagnosis were both 6.8 cm (range: 2.8–11.6 cm; Figure 2D). Five (50%) were described by the pathologist as epithelioid and spindle cell rhabdomyosarcoma, three (30%) were described as a spindle cell/sclerosing rhabdomyosarcoma, one (10%) as a high-grade rhabdoid malignant tumor, and one (10%) as a small-cell sarcoma with rhabdomyogenic/rhabdomyosarcoma differentiation. These tumors were highly proliferative; five had at least 10 mitoses per 10 high-powered fields (hpf) and one had Ki67 of >80%. Most or all were positive for ALK (Figure 2E), cytokeratins (Figure 2F), desmin (Figure 2G), MyoD1 (Figure 2H), and myogenin (Figure 2I).

Six patients (60%) presented with localized disease and four (40%) with de novo metastatic disease (Figure 2J). Most patients developed locally recurrent disease (5/10, 50%). Lymph node metastases were reported in half (5/10, 50%) of the patients in our cohort (Table 1).

### 3.2. Treatment

**Figure 3:**
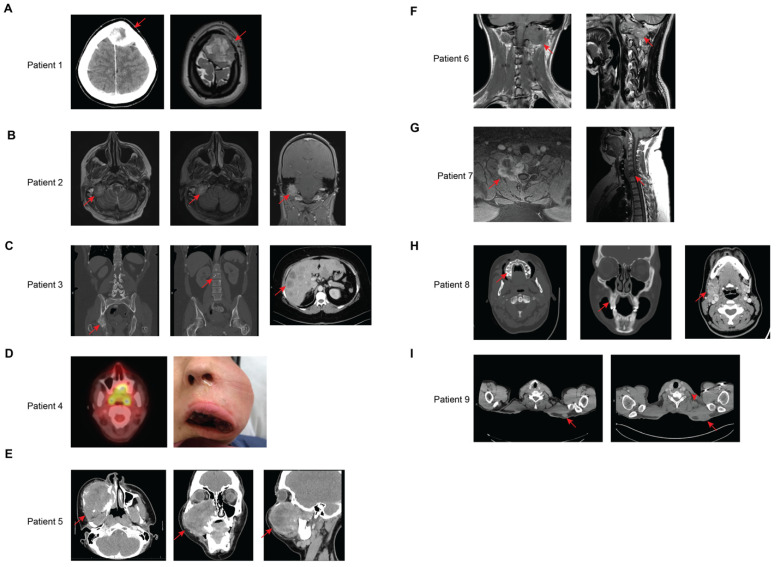
Patients with *TFCP2* fusion sarcomas. (**A**) Patient 1, CT (left) and MRI (right) images from initial diagnosis showing left frontal bone tumor. (**B**) Patient 2, MRI demonstrating involvement of the right IJV and right ICA and extension into the posterior cranial fossa. (**C**) Patient 3, CT images at the time of diagnosis showing presumed primary tumor in right acetabulum (left), additional diffuse spinal metastases (middle), and diffuse hepatic metastases (right). (**D**) Patient 4, PET/CT showing primary FDG-avid mass of the hard palate at the time of diagnosis (left) and an image of the patient after development of recurrent disease (right). (**E**) Patient 5, CT images showing large tumor in axial (left), coronal (middle), and sagittal (right) planes. (**F**) Patient 6, sagittal (left) and coronal and sagittal (right) MRI images showing mass arising from C1. (**G**) Patient 7, axial and sagittal MRI images of mass arising from C7. (**H**) Patient 8, CT images of mass arising in right alveolar ridge in bone window (left, middle) and lymphadenopathy in soft tissue window (right). (**I**) Patient 9, CT at time of diagnosis (left) and local recurrence after surgery (right) with cervical lymphadenopathy (arrowhead). Red arrows highlight location of the tumors.

Treatment and imaging data were available for 9 of the 10 patients. The first patient, a 21-year-old woman with *TFCP2* fusion sarcoma of the left frontal bone (Figure 3A) was initially treated with surgery, developed local recurrence with invasion of the underlying brain parenchyma approximately 2 months later and underwent radiation (VMAT 64.2 Gy in 30 fractions). Three months later, she developed distant recurrence in the bilateral lungs and was treated with chemotherapy with VDC, although she developed progressive disease after two cycles. She was then treated with irinotecan plus temozolomide and had a mixed response, with response in the lungs but progression in the brain. The patient died shortly after.

The second patient is a 35-year-old male with *EWSR1-TFCP2* fusion sarcoma of the right occipital bone with extension into the posterior cranial fossa and involvement of the right internal jugular vein and right internal carotid artery (Figure 3B). He was treated with gemcitabine plus docetaxel and achieved a mixed response. The next-generation sequencing panel showed an ALK inversion, so he was subsequently treated with lorlatinib; this was switched to alectinib after 1 week due to his insurance no longer covering lorlatinib. The patient initially had a positive response, and the patient’s symptoms also improved. However, after about 2 months he developed progressive disease. He was then treated with vincristine plus doxorubicin plus ifosfamide (VAI) and developed progressive disease after two cycles, with widespread metastases to the bilateral lungs and axial and appendicular skeleton. He was then treated with vincristine plus irinotecan plus temozolomide for four cycles, with an initial positive response after two cycles, but then progressed after four cycles. The patient died shortly thereafter.

The third patient is a 48-year-old female with *FUS-TFCP2* fusion sarcoma that likely originated in the right hip but presented with de novo metastatic disease to the bilateral lungs, liver, lymph nodes, and bone (Figure 3C). She was treated with gemcitabine plus docetaxel but developed progressive disease after two cycles. She then received VDC for a total of five cycles, with a mixed response. She relocated and transitioned her care to another cancer center, although she died approximately 2 months later.

The fourth patient is a 30-year-old female with *FUS-TFCP2* fusion sarcoma of the hard palate (Figure 3D). This was resected, and she subsequently developed local recurrence. She was treated initially with vincristine plus actinomycin D plus cyclophosphamide but stopped after one cycle due to poor tolerance. She was then treated with single-agent actinomycin D but only received one cycle again due to poor tolerance to therapy. She developed local progression and received three additional cycles of VAC but stopped due to toxicity. She was administered 60.4 Gy in 28 fractions to the primary tumor. Unfortunately, her cancer continued to progress, and she was unable to tolerate further therapy and passed away 4 months after completing radiation.

The fifth patient is a 13-year-old male with *FUS-TFCP2* fusion sarcoma of the right maxilla (Figure 3E). He was treated with neoadjuvant VAC, although he developed progressive disease after 10 weeks and was switched to VDC/IE as per the Children’s Oncology Protocol, ARST0431, which achieved a partial response. He received radiation (41.4 Gy to right maxilla and neck with 9 Gy boost). After the completion of planned therapy, he continued to have gross disease, and he underwent resection with right total maxillectomy, right total ethmoidectomy, sphenoidotomy, frontal sinusotomy, right infratemporal fossa dissection, and fibula free flap reconstruction. Imaging 2 months following surgery showed concern for local recurrence, with cutaneous and subcutaneous nodules in the neck and periorbital area. He was started on lorlatinib and achieved a partial response after 3 months of treatment, with complete resolution of many of the subcutaneous and cutaneous nodules along the lateral aspect of the right face. He developed progressive disease after 3 more months and was switched to entrectenib, although he developed progressive disease and worsening pain after one cycle. With no viable treatment options and given that lorlatinib appeared to have slowed disease progression, lorlatinib was restarted, and he was treated with this for about 6 months before further progression. He is now receiving comfort-focused care.

The sixth patient is an 11-year-old girl that presented with left-sided neck pain and was found to have a sarcoma arising from C1 that had extended locally to the vertebral arteries and spinal cord (Figure 3F). She was treated with VAC alternating with vincristine and irinotecan but developed progression after two cycles. She received 50.4 Gy of radiation to the tumor, which did result in a decrease in the size of the tumor. She was then treated with lorlatinib for about one month, although the disease progressed rapidly during this time, and the patient died shortly afterwards.

The seventh patient is a 35-year-old man with tumor arising from paraspinal muscles near C7 (Figure 3G). He was treated with neoadjuvant vincristine, doxorubicin, and cyclophosphamide but stopped after restaging imaging after two cycles showed progressive disease. He then received radiation with 50 Gy to the tumor and was then started on crizotinib and subsequently underwent surgical resection. Crizotinib was held during the perioperative window and resumed. Three months later, he developed recurrent disease locally and new lung metastases.

The eighth patient (Figure 3H) is a 33-year-old woman that developed a sarcoma of the right alveolar ridge. PET showed FDG-avid right cervical lymphadenopathy. She was treated with neoadjuvant vincristine, dactinomycin, and cyclophosphamide for four cycles. Restaging imaging showed progressive disease and new metastases in the lungs. She was then treated with single-agent ifosfamide (10 g/m^2^) with concurrent radiation for pain control and received two cycles before developing progressive disease at the first restaging imaging. She was then treated with ipilimumab and nivolumab and received two cycles, at which point first restaging imaging showed progression in the head and neck, lymph nodes, lung, liver, and bone. One of the sites of disease was encasing and starting to narrow the right internal carotid, and radiation was planned. However, the patient declined and died quickly.

The ninth patient is a 40-year-old man who presented with a mass in his upper back (Figure 3I). He was treated with VAC for 13 weeks and then underwent wide local excision. Two weeks later, he developed local recurrence and resumed VAC. He had further progression and was switched to VDC. He was started on a clinical trial with local injection of an oncolytic herpes virus but developed progressive disease after three treatments.

**Figure 4:**
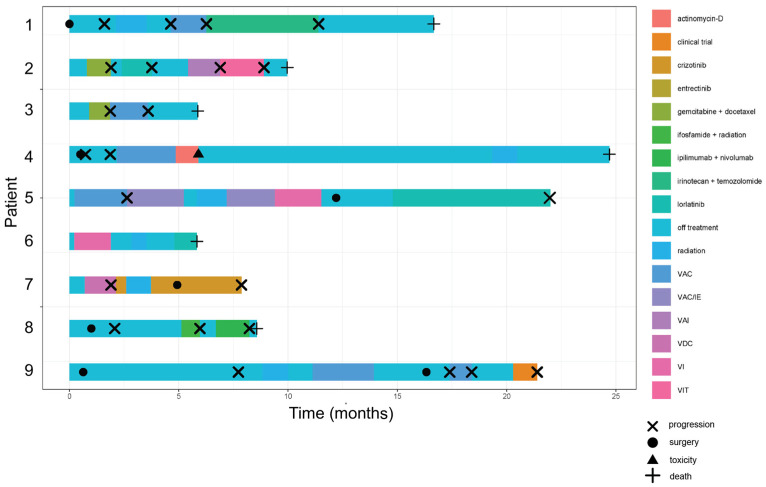
Treatment of *TFCP2* fusion sarcomas. Swimmer’s plot showing timeline of indicated treatments for 9 out of 10 patients for which treatment data were available. Time 0 is the date of diagnosis. VAC = vincristine, actinomycin, and cyclophosphamide. VAI = vincristine, doxorubicin, and ifosfamide. VDC = vincristine, doxorubicin, and cyclophosphamide. VI = vincristine and ifosfamide. VIT = vincristine, ifosfamide, and temozolomide.

A swimmer’s plot summarizing the treatment of each patient is shown in Figure 4. This demonstrates the timing of each treatment and the reason for changing treatment (progression or toxicity).

### 3.3. Clinical Outcomes

**Figure 5:**
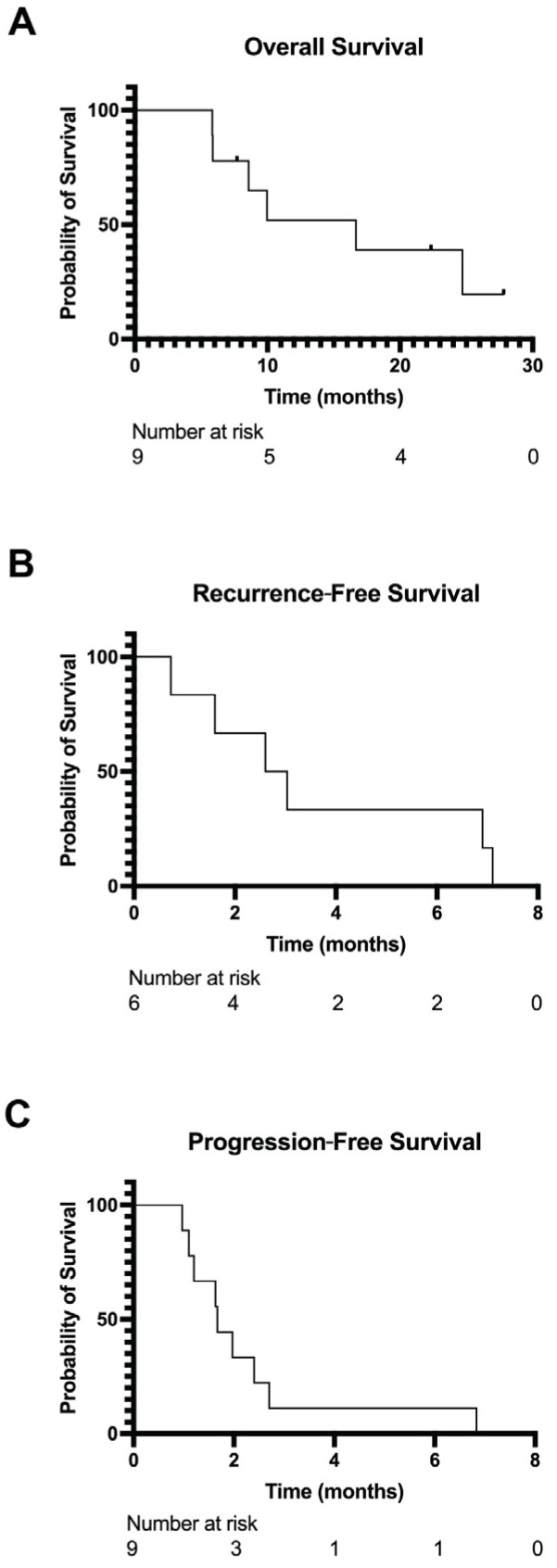
Survival outcomes in *TFCP2* fusion sarcomas. (**A**) Overall survival of the patients with available treatment data (*n* = 9). (**B**) Recurrence-free survival was calculated in patients with initially localized disease from the date of histologic diagnosis to the date of recurrence, death, or the latest follow-up (*n* = 6). (**C**) Progression-free survival of the patients with available treatment data was calculated from the start of systemic therapy for recurrent or metastatic disease to the date of progression, death, or the latest follow-up (*n* = 9).

The median overall survival in this cohort was 16.7 months (range: 5.9–24.7 months after diagnosis; Figure 5A). Six patients were treated with upfront surgery, and all six developed recurrent disease. The median time to recurrence following upfront surgery was 2.8 months (range: 0.73–7.1 months; Figure 5B). Nine patients received systemic therapy, and the median progression-free survival from the start of treatment to progression was 1.7 months (range: 0.97–6.8; Figure 5C).

### 3.4. Review of the Literature

**Figure 6:**
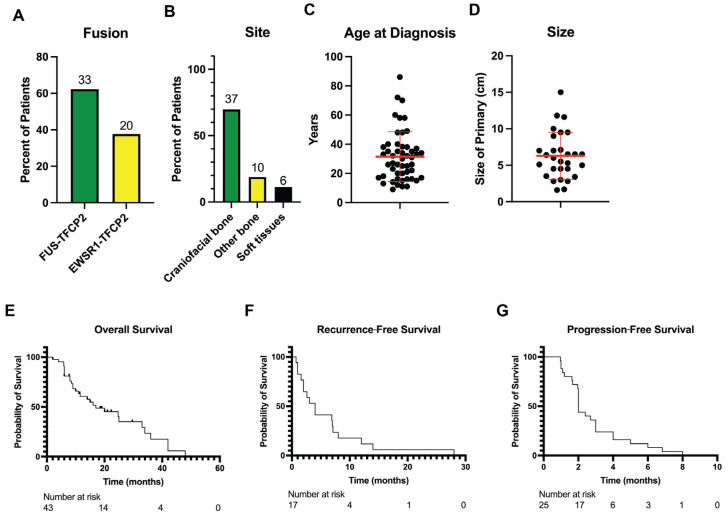
Summary of 53 cases of *TFCP2* fusion sarcomas in the literature. (**A**) Percent of patients with each of the two *TFCP2* fusions, either *FUS-TFCP2* or *EWSR1-TFCP2*. (**B**) Breakdown of the site of disease. (**C**) Distribution of the age at diagnosis. (**D**) Distribution of the size of the sarcoma at diagnosis. In (**C**,**D**), bars represent means ± standard deviations, and each black dot represents a different patient. (**E**) Overall survival of the cohort (*n* = 43). (**F**) Recurrence-free survival was calculated in patients with initially localized disease from the date of histologic diagnosis to the date of recurrence, death, or the latest follow-up (*n* = 17). (**G**) Progression-free survival of the cohort was calculated from the start of systemic therapy for recurrent or metastatic disease to the date of progression, death, or the latest follow-up (*n* = 25).

In reviewing the literature, we identified 53 cases of *TFCP2* fusion sarcomas, including the 10 reported herein (Table 2). These were slightly more common in females (56.6%). The *FUS-TFCP2* fusion was present in 63.0%, and the *EWSR1-TFCP2* fusion was present in 37.0% (Figure 6A). There was a predilection for craniofacial bones (69.8%; Figure 6B). The tumors less commonly arose from other bony sites (18.9%) and soft tissues (11.3%). The mean and median ages of diagnosis were 31.5 and 30 years, respectively (Figure 6C). There was one outlier in a 72-year-old woman [16]. The mean and median tumor sizes were 6.3 cm and 6 cm, respectively (Figure 6D). Other reported alterations included *ALK* overexpression as well as *ALK* intragenic deletions and aberrant splicing and *CDKN2A* and *MTAP* deletions (Table 2) [15]. Regarding outcomes, the median overall survival for the 43 cases with reported outcomes was 17 months (Figure 6E). For the 17 cases with localized disease at presentation with reported outcomes, the median recurrence-free survival was 4 months (range: 0.73–28 months; Figure 6F). For the 25 cases with metastatic disease treated with systemic therapy and reported outcomes, the median progression-free survival was 2 months (range: 0.96–8 months; Figure 6G).

We assessed for potential differences in patient and tumor characteristics and outcomes based on the fusion partner (e.g., *EWSR1* or *FUS*). Tumors with the *EWSR1-TFCP2* fusion may be slightly more likely to arise from soft tissues than those with *FUS-TFCP2* (Supplemental Figure S1A). There was no difference in fusion partner based on the patient gender or sex, age at diagnosis, or size of primary tumor. Furthermore, there were no significant differences in patient outcomes based on the fusion partner (Supplemental Figure S1).

## 4. Discussion

Spindle cell/sclerosing rhabdomyosarcomas typically harbor certain genomic alterations, including rearrangements involving *TFCP2*, *VGLL2*, *NCOA2*, *CITED2*, *TEAD1*, and *SRF*, as well as the *MYOD1 p.L122R* mutation [17,18,19]. There are also rarer molecular events that have been reported, such as a *RAB3IP-HMGA2* fusion [20]. In the present study, we sought to understand the clinical characteristics, efficacy of treatments, and clinical outcomes in patients with *TFCP2* fusion sarcomas. Analysis of the 10 cases from our institution along with the additional 43 cases identified in the literature allowed us to more comprehensively assess the clinical characteristics and outcomes of this orphan disease. In general, we find that *TFCP2* fusion often results in an epithelioid and spindle cell rhabdomyosarcoma that most commonly affects young women. Sarcomas do not typically involve lymph nodes, with some exceptions in specific subtypes, such as epithelioid clear cell, angiosarcoma, synovial sarcomas, and rhabdomyosarcomas. However, several cases of *TFCP2* fusion sarcoma reported in the literature have noted lymph node involvement [13,15], and 5 of the 10 patients described herein had lymph node involvement. Unfortunately, outcomes for this disease are extremely poor, leading to significant loss of life-years in this young population. More than half of patients with localized disease developed recurrent disease. Assessing for the fusion is critical for the diagnosis, as we encountered multiple cases in the literature that were initially misclassified as a different malignancy, including DSRCT [8].

A recent study performed multi-omics analysis of 12 cases of RMS with *TFCP2* fusion to better understand the biology of this disease [15]. Six out of eleven patients with localized disease eventually developed distant metastases, mostly in the lungs and lymph nodes. Six patients received ALK inhibitors, and their responses were poor. RNA sequencing of these tumors was notable for elevated ALK expression. Exogenous expression of the ALK variants seen in *TFCP2* fusion sarcomas in p53-deficient MCF10A untransformed human breast epithelial cell lines is sufficient for transformation. Furthermore, these alterations sensitized cells to ALK inhibitors, namely ceritinib. However, exogenous expression of the *TFCP2* fusion is not sufficient for transformation but does block myogenic differentiation. This study also found that many *TFCP2* fusion sarcomas have *CDKN2A* loss. Interestingly, the genomes were quite complex and showed a homologous recombination deficiency signature, which is unusual for fusion positive sarcoma and was more reminiscent of undifferentiated complex karyotype sarcomas. Therefore, immunotherapy may be worth trialing in this disease, although enthusiasm should be quelled by the limited efficacy of immunotherapy in other sarcomas with complex karyotypes, such as leiomyosarcoma [21].

What is the optimal way to treat *TFCP2* fusion sarcomas? Unfortunately, these tumors have shown limited sensitivity to conventional chemotherapies. ALK alterations, namely inversions and intragenic deletions, as well as ALK protein overexpression seen via IHC, commonly co-occur with *TFCP2* fusion [15], and the upregulation of ALK activity leads to increased cell proliferation and migration in multiple cancer types [22]. The development of targeted therapies with ALK inhibitors has shown positive outcomes in many tumor types, including other sarcomas such as inflammatory myofibroblastic tumors, where ALK inhibitors have shown benefits [23,24,25,26]. Despite the prevalence of *ALK* alterations and overexpression, most *TFCP2* fusion sarcoma patients treated with ALK inhibitors have not responded well. One patient treated with crizotinib progressed rapidly after 10 days of treatment [27]. One patient in our study progressed rapidly on lorlatinib. However, one patient in our cohort did have a near complete response, and the total duration of clinical benefit with lorlatinib was about 6 months. Future work will need to focus on the mechanisms of resistance to ALK inhibition. Borrowing from the lung cancer literature, where ALK alterations are more commonly seen, the mechanisms of resistance to ALK inhibitors include acquired resistance mutations in *ALK*; *ALK* gene amplification; the activation of downstream or bypass signaling pathways, including *EGFR*, *MET*, and *MAPK*; and the expression of drug efflux pumps [28,29,30,31]. These mechanisms were largely discerned with genomic profiling following resistance. However, these data are lacking in *TFCP2*-fusion sarcomas and are a critical area of future investigation.

Other potential ideas for future trials in this disease may come from studying the concurrent genetic aberrations, namely *CDKN2A* and *MTAP* co-deletion [15]. *CDKN2A* is a known tumor suppressor, and its loss may render sensitivity to CDK4/6 inhibitors. *MTAP* is a gene adjacent to *CDKN2A* on chromosome 9p and is frequently co-deleted with *CDKN2A* in a wide range of cancers. *MTAP* is an enzyme involved in the salvage of methionine and adenine. Preclinical studies identified that PRMT5 inhibition is synthetic lethal with *MTAP* loss [32]. Furthermore, *MTAP* loss also renders sensitivity to antifolates such as pemetrexed. In a single-arm phase 2 trial in urothelial carcinoma, 3 out of 7 patients showed a response to pemetrexed (ORR 43%). In addition, analysis of a historic cohort showed 4 out of 4 *MTAP*-deficient tumors responded to pemetrexed compared to 1/10 MTAP proficient [33]. Therefore, PRMT5 inhibitors and antifolate drugs may be potential therapeutic options to pursue in *TFCP2* fusion sarcomas with *MTAP* deletion. Furthermore, given the reported homologous recombination deficiency signature seen [15], this may suggest trialing platinum-based chemotherapy or DNA damage pathway inhibitors such as PARP inhibitors. Lastly, small molecules have been developed that may interfere with *TFCP2* binding to DNA [34,35] and may be of therapeutic interest.

There are several limitations to our study. Due to the rarity of this disease, we have a small sample size, which limits the ability to make statistically significant conclusions, although to mitigate this, we incorporated analyses of all previously published cases. This disease does not have a standardized treatment regimen and, thus, provides another layer of complexity in comparing responses to treatments, as each patient often has a unique and personalized treatment course. Next, there is potential bias in patient selection, as we included only patients seen at our institution, a large tertiary referral center.

## 5. Conclusions

In summary, we report our experience with 10 cases of *TFCP2* fusion sarcomas and review the literature of 53 total cases. This particular subtype of sarcoma is associated with a dismal prognosis in a young population, resulting in significant loss of life-years. Our clinical data may serve as a benchmark against the efficacy of future novel therapies. ALK inhibitors have been largely unsuccessful, although one patient at our institution derived significant clinical benefit from lorlatinib. There is great need to develop novel treatment strategies for this orphan disease, as well as further investigate the genetic profiles of these tumors to determine prognostic factors, oncogenic drivers, and directed therapy targets.

## Figures and Tables

**Table 1:** Clinical characteristics of patients with TFCP2 fusion sarcomas.

ID	Age of Sarcoma Diagnosis	Sex	Fusion	Pathology	Site	Desmin	MyoD1	ALK	Myogenin	Cytokeratin	Size of Primary Tumor (cm)	Stage	Site(s) of Metastases	Mitoses	Primary Surgery	Chemo 1st Line	Best Response	Chemo 2nd Line	Best Response	Radiation?	Radiation Summary	How Was Fusion Discovered?	Other Mutations	Personal History of Cancer	Family History of Cancer
1	21	Female	FUS-TFCP2	Spindle cell rhabdomyosarcoma	Left frontal bone	Positive	Positive	Positive	Positive	Positive	7	T3 N0 M0	Brain parenchyma underlying primary site, lungs	>20/10 hpf	Yes	VDC	SD	Irinotecan + temozolomide	Mixed	Yes	64.2 Gy in 30 fx	Mayo Clinic fusion panel	NA	None	None
2	35	Male	EWSR1-TFCP2	High-grade rhabdoid malignant tumor	Right occipital skull base (cerebellopontine angle)	Positive	Rare	Positive	Rare	Positive	2.8	T2 Nx M1	Lymph nodes, and lungs	Ki-67 > 80%	No	Gem/docetax	Mixed	Lorlatinib, alecitinib (switched from lorlatinib after 5 days due to insurance issue)	SD	No	N/A	NGS panel	CDKN2A and CDKN2B loss, ALK inversion	None	None
3	48	Female	FUS-TFCP2	Poorly differentiated epithelioid and spindle cell rhabdomyosarcoma	Right acetabulum	Positive	Positive	Positive	Negative	Positive	11.6	IV (T3 Nx M1)	Lungs, liver, lymph nodes, and bones	Unknown	No	Gem/docetax	PD	VDC	Mixed	No	N/A	Mayo Clinic fusion panel	NA	None	Paternal grandmother and great-grandmother with stomach cancer
4	30	Female	FUS-TFCP2	Epithelioid and spindle cell rhabdomyosarcoma	Hard palate	Positive	Positive	Positive	Positive	Positive	3.5	T2 N0 M0	Locally	10/10 hpf	Yes	VAC	PD	Single-agent actinomycin-D	PD	Yes	60.4 Gy in 28 fx	Mayo Clinic fusion panel	NA	None	Maternal grandma with liver; maternal uncle with lung
5	13	Male	FUS-TFCP2	Small-cell sarcoma with rhabdomyogenic/rhabdomyosarcoma differentiation	Right maxilla	Positive	Positive	Positive	Very rare	Very rare	9.5	Group III, Stage 3 (pediatric)	Lymph nodes, and lungs	4/10 hpf	Yes	VAC	Mixed	VDC/IE	PR	Yes	50.4 Gy in 28 fx	Mayo Clinic fusion panel	NA	None	Unspecified cancer in paternal grandfather
6	11	Female	FUS-TFCP2	High-grade rhabdomyosarcoma with spindle and epithelioid cells	C1	Positive	Positive	Positive	Positive	Positive	7.1	IV (T2b, N0, M1)	Local extension to vertebral arteries, spinal cord on presentation	10/10 hpf	No	Vincristine, irinotecan	PD	VI (with concurrent radiation)	PR	Yes	50.4 Gy in 28 fx concurrently with VI	NGS panel	NA	None	None
7	35	Male	FUS-TFCP2	Spindle cell sarcoma with rhabdomyosarcomatous differentiation	C7 paraspinal musculature	Positive	Positive	Positive	Positive	Positive	4.5	cT1, cN0, cM0	Pulmonary mets present on presentation, enlarged on subsequent scans	8/10 hpf	No	VDC	PD	Crizotinib	PD	Yes	50 Gy in 25 fx	NGS panel	NA	None	Maternal grandma with breast cancer
8	33	Female	EWSR1-TFCP2	Spindle cell sclerosing rhabdomyosarcoma	Right maxilla	Positive	Positive	Positive	Positive	Positive	1.7	cT4, cN1, cM0	Local nodal spread and small pulmonary nodules on presentation	8/10 hpf	Yes	VDC	PD	Lorlatinib + ifosfamide	PD	Yes	36 Gy in 12 fx	NGS panel	NA	None	Maternal grandfather with unspecified cancer
9	40	Male	EWSR1-TFCP2	Epithelioid and spindle cell rhabdomyosarcoma	Left trapezius mass	Positive	Positive	Positive	Positive	Positive	6.5	Stage IV (cT2, cN1, cM0)	C5 LAD	10/10 hpf	Yes	VAC	SD	VAC (resumed post-surgery)	PD	Yes	50 Gy in 25 fx	NGS panel	NA	None	Father with skin cancer; paternal aunt with lung cancer
10	34	Male	FUS-TFCP2	High-grade rhabdomyosarcoma with epithelioid and spindle cell features	Mandible and floor of mouth	Positive	Positive	Not tested	Not tested	Not tested	6.5	T3 N0 M0	Locally	10/10 hpf	Yes	Unknown	NA	Unknown	NA	Unknown	NA	NGS panel	NA	None	None

NA: not applicable or available.

**Table 2:** Review of the literature of TFCP2 fusion sarcoma cases.

Study Author	PMID	Fusion	Sarcoma Subtype, Pathology	Co-Occurring Molecular Alterations	Desmin	MyoD1	ALK	Myogenin	Cytokeratin	Location	Size (cm)	Mitotic rate	Age at Sarcoma Diagnosis	Sex	Personal History of Other Cancers	Treatment Summary	Surgery?	Chemo?	Radiation?	Overall Outcome	Time to Local Recurrence	Time to Progression	Time to Distant Recurrence	Follow Up
Ginn	NA	FUS-TFCP2	Spindle cell rhabdomyosarcoma	NA	Positive	Positive	Positive	Positive	Positive	Left frontal bone	7	>20/10 hpf	21	Female	None	Surgery, local recurrence; radiation, distant recurrence; VDC, progression; irinotecan/temozolomide, progression, death	Yes	VDC, irinotecan/temozolomide	64.2 Gy in 30 fx	DOD	1.6 mo	1.6 mo	4.6 mo	16.7 mo
Ginn	NA	EWSR1-TFCP2	High-grade rhabdoid malignant tumor	CDKN2A and CDKN2B loss, ALK inversion	Positive	Rare	Positive	Rare	Positive	Right occipital skull base (cerebellopontine angle)	2.8	Ki-67 > 80%	35	Male	None	Gem/docetaxel, mixed response; lorlatinib/alectinib, progression; VAI, progression; VIT, progression, death	No	Gem/docetax, lorlatinib/alectinib, VAI	N/A	DOD	NA	1.1 mo	1.9	10.0 mo
Ginn	NA	FUS-TFCP2	Poorly differentiated epithelioid and spindle cell rhabdomyosarcoma	NA	Positive	Positive	Positive	Negative	Positive	Right acetabulum	11.6	Unknown	48	Female	None	Gem/docetaxel, progression; VDC, mixed response, death	No	Gem/docetax, VDC	N/A	DOD	NA	1.0 mo	0	5.9 mo
Ginn	NA	FUS-TFCP2	Epithelioid and spindle cell rhabdomyosarcoma	NA	Positive	Positive	Positive	Positive	Positive	Hard palate	3.5	10/10 hpf	30	Female	None	Surgery, local recurrence; VAC, acinomycin, progression; radiation, progression, death	Yes	VAC, actinomycin D	60.4 Gy in 28 fx	DOD	0.7 mo	2.7 mo	NA	24.7 mo
Ginn	NA	FUS-TFCP2	Small-cell sarcoma with rhabdomyogenic/rhabdomyosarcoma differentiation	NA	Positive	Positive	Positive	Very rare	Very rare	Right Maxilla	9.5	4/10 hpf	13	Male	None	VAC, progression; VDC/IE, PR, radiation, surgery, local recurrence; lorlatinib, progression; entrectenib, progression, lorlatinib	Yes	VAC, VDC/IE	50.4 Gy in 28 fx	AWD	6.9	NA	6.9	27.8 mo
Ginn	NA	FUS-TFCP2	High-grade rhabdomyosarcoma with epithelioid and spindle cell features	NA	Positive	Positive	Not tested	Not tested	Not tested	Mandible and floor of mouth	6.5	10/10 hpf	34	Male	None	NA	Yes	NA	NA	NA	6.9 mo	NA	6.9 mo	6.9 mo
Ginn	NA	FUS-TFCP2	HIGH-GRADE RHABDOMYOSARCOMA WITH SPINDLE AND EPITHELIOID CELLS	NA	Positive	Positive	Positive	Positive	Positive	C1	7.1	10/10 hpf	11	Female	None	Vincristine/irinotecan, progression after 2 cycles; radiation, PR, lorlatinib, progressed after 1 month, death	No	Vincristine, irinotecan	50.4 Gy in 28 fx concurrently with VI	DOD	NA	1.7 mo	NA	5.8 mo
Ginn	NA	FUS-TFCP2	Spindle cell sarcoma with rhabdomyosarcomatous differentiation	NA	Positive	Positive	Positive	Positive	Positive	C7 paraspinal musculature	4.5	8/10 hpf	35	Male	None	Neoadjuvant VDC, progression after 2 cycles; radiation, crizotinib, resection, continued crizotinib, local and distant recurrence 3 months after resection	No	VDC	50 Gy in 25 fx	AWD	NA	1.2 mo	NA	7.7 mo
Ginn	NA	EWSR1-TFCP2	Spindle cell sclerosing rhabdomyosarcoma	NA	Positive	Positive	Positive	Positive	Positive	Right maxilla	1.7	8/10 hpf	33	Female	None	VAC, local and distant progression after 4 cycles; ifosfamide, progression after 2 cycles; ipilimumab/nivolumab, progressed after 2 cycles, death	Yes	VDC	36 Gy in 12 fx	DOD	3.0 mo	2.0 mo	NA	8.6 mo
Ginn	NA	EWSR1-TFCP2	Epithelioid and spindle cell rhabdomyosarcoma	NA	Positive	Positive	Positive	Positive	Positive	Left trapezius mass	6.5	10/10 hpf	40	Male	None	VAC, resection, local progression; VAC, progression; VDC, progression; clinical trial, progression	Yes	VAC	50 Gy in 25 fx	AWD	7.1 mo	6.8 mo	NA	22.3 mo
Watson	29431183	EWSR1-TFCP2	Desmoplastic small-round-cell tumor (prior to sequencing)	MCFD2-PREPL; POLA2-TCIRG1; TFCP2-BRDT; CAND2-PPARG; ZNF584-TBC1D30						Chest wall			38.3	F		Avg survival 5.5 months								
Watson	29431183	FUS-TFCP2	Rhabdomyosarcoma							Pelvic bone			26.1	F										
Watson	29431183	FUS-TFCP2	Osteosarcoma							Sphenoid bone			16.1	F										
Chrisinger	32556562	EWSR1-TFCP2	Spindle–epithelioid cell rhabdomyosarcoma		Pos		Pos	Neg	Neg	Right frontal bone	5 × 4.6 × 4 cm	>20/10 hpf	mid 20 s–30 s	F		VDC x 3 cycles: no change; concurrent radiation with another 2 cycles, with decrease in size, went for resection; resulted in wound dehiscence and infection, multiple OR visits with 2 skin flaps, unable to continue chemo for 4 months; at 8-month scan had mets to R acetabulum and L iliac; continued to have growth of mets, expired from disease 17 months after diagnosis	Removal of mass following chemo-XRT	VDC × 3 cycles	50.4 Gy in 28 fx	DOD	8 mo		8 mo	17 mo
Chrisinger	32556562	FUS-TFCP2	Rhabdomyosarcoma (epithelioid, spindle, and rhabdoid)		Pos	Pos	Pos	Pos		Pelvic bone	9.5 cm	25/10 hpf	20s	F		Multi-agent chemotherapy × 2 months with overall tumor burden reduction; repeat scans in 3 months with mixed response, concern for progression in main tumor radiation of primary site with VI; continued on high-risk RMS protocol for 3 months; stable scans 3 months later, progression with new mets; started on temsirolimus, vinorelbine, and cyclophosphamide; passed 6 weeks later, 11 months after diagnosis	None	High-risk RMS protocol (Ifosfamide, vincristine, etoposide, doxorubicin, cyclophosphamide, and dactinomycin) Temsirolimus, vinorelbine, cyclophosphamide	50.4 Gy in 28 fx	DOD		3 mo		11 mo
Dashti	29758589	FUS-TFCP2	Spindle cell RMS		Pos	Pos	Pos	Pos		Mandible	2.8 cm		72	M		Resection of primary tumor, declined adjuvant chemo	Resection	Declined		ANED				2 mo
Lewin	31470995	FUS-TFCP2	Rhabdomyosarcoma	PBRM1 loss (associated with PD-1 and CTLA-4/PD-1 checkpoint inhibitors; did not target due to poor ECOG status)		Pos	Pos	Pos		Nasal cavity			23	M		Rapid progression through 4 cycles of anthracycline-based chemo + external beam radiation to primary sinonasal tumor; transitioned to crizotinib 250 mg BID, stable disease after 4 weeks, died 3 weeks later, 2/2 symptomatic pleural effusion	None	Anthrocycline-based chemo for 4 cycles crizotinib 250 mg BID for ~7 weeks	Radiation to primary site, unknown amount or fx	DOD		4 mo		6 mo
Agaram	30720533	EWSR1-TFCP2	Spindle–epithelioid cell rhabdomyosarcoma		Pos	Pos	Pos	Pos	Pos	Skull		>15/10	27	F		Unknown				Unknown				Unknown
Agaram	30720533	EWSR1-TFCP2	Spindle–epithelioid cell rhabdomyosarcoma		Pos	Pos	Pos	Pos	Pos	Maxilla, masticator space, sinuses, orbit, and clivus; mets to femur		>15/10	33	F		Unknown				Unknown				Unknown
Agaram	30720533	EWSR1-TFCP2	Spindle cell RMS		Pos	Pos	Pos	Pos	Pos	Femur		>15/10	20	M		Unknown				Unknown				Unknown
Agaram	30720533	FUS-TFCP2	Spindle cell RMS		Pos	Pos	Pos	Pos	Pos	Iliac		<15/10	37	F		Unknown				Unknown				Unknown
Tagami	30948206	FUS-TFCP2	Spindle cell RMS		Pos	Pos	Pos	Pos	Pos	Lumbar vertebra		12/10 hpf	70	F		Surgery --> docetaxel + XRT (30 Gr/10fr + 21 Gr/7fr) --> adriamycin				AWD				6 mo
Le Loarer	31383960	FUS-TFCP2	Spindle–epithelioid cell rhabdomyosarcoma		Pos	Pos	Pos	Pos	Pos	Sphenoid bone	9	12	16	F		4 cycles etop, ifos --> resection --> local relapse 2 mo later with distant mets --> 1 cycle cyclophos and doxo + XRT --> progression --> death				DOD	2 mo	4 mo	2 mo	15 mo
Le Loarer	31383960	FUS-TFCP2	Epithelioid cell RMS		Pos	Pos	Pos	Pos	Pos	Sacrum	10	18	26	F		2 cycles IVADO --> progression --> 1 cycle IVE --> progression --> 1 cycle VAI				DOD		2 mo		4 mo
Le Loarer	31383960	EWSR1-TFCP2	Spindle–epithelioid cell rhabdomyosarcoma		Pos	Pos	Pos	Pos	Pos	Peritoneum		12	38	F		1 cycle carboplatin, paclitaxel --> progression --> 1 cycle adriamycin, holoxan --> lymphangitic carcinomatosis, death				DOD		1 mo		2 mo
Le Loarer	31383960	EWSR1-TFCP2	Spindle–epithelioid cell rhabdomyosarcoma		Pos	Pos	Neg	Neg	Pos	Hard palate and upper lip	3	21	32	M		3 cycles VAI --> local progression --> 1 cycle etoposide–carboplatin --> local progression --> 1 cycle actinomycin–cyclophosh --> local progression				DOD		3 mo		8 mo
Le Loarer	31383960	FUS-TFCP2	Spindle–epithelioid cell rhabdomyosarcoma		Pos	Pos	Neg	Pos	Pos	Orbito-temporal-sphenoid	15 x 14.5	32	20	M		1 cycle doxo-ifos --> local progression --> 3 cycles VAC --> local progression --> XRT + pazopanib --> local progression				DOD		1 mo		6 mo
Le Loarer	31383960	EWSR1-TFCP2	Spindle–epithelioid cell rhabdomyosarcoma		Pos	Pos	Pos	Pos	Pos	Inguinal	6.5 x 4.0	10	86	M		Resection --> recurrence 4 mo --> palliative --> progressed in 2 mo				DOD	4 mo	2 mo		6 mo
Le Loarer	31383960	EWSR1-TFCP2	Spindle–epithelioid cell rhabdomyosarcoma		Pos	Pos	Pos	Pos	Pos	Femur	5.1	8	18	F		2 cycles VDC IE --> local progression --> pazopanib --> local progression				DOD		2 mo		8 mo
Le Loarer	31383960	FUS-TFCP2	Epithelioid cell RMS		Pos	Pos	Pos	Neg	Pos	Cervico-occipital	5.3 × 3.7 × 4.4	16	17	F		2 cycles VDC IE + XRT (60 Gy) --> local progression --> crizotinib 500 mg/d for 1 month then switched to alectinib 1200 mg/d --> stable disease x 9 mos				AWD		8 mo		15 mo
Le Loarer	31383960	FUS-TFCP2	Spindle–epithelioid cell rhabdomyosarcoma		Pos	Pos	Pos	Pos	Pos	Left occipital	11.8	66	31	M		Resection --> relapse at 1 mo --> 3 cycles AI --> local progression and distant mets --> death				DOD	1 mo	3 mo	3 mo	6 mo
Le Loarer	31383960	FUS-TFCP2	Spindle cell RMS		Pos	Pos	Pos	Pos	pos	Mandible	4.5 × 3.2	21	32	M		Surgery + adjuvant 5 cycles doxo + ifos --> local relapse at 12 mo + distant met --> 3 cycles gemcitabine, docetaxel				AWD	12 mo		12 mo	14 mo
Le Loarer	31383960	FUS-TFCP2	Spindle–epithelioid cell rhabdomyosarcoma		Pos	Pos	Pos	Pos	Pos	Mandible	1.6 × 1.5 × 1.3	11	58	F		Surgery + adjuvant CT + XRT --> NED				ANED				21 mo
Le Loarer	31383960	FUS-TFCP2	Spindle–epithelioid cell rhabdomyosarcoma		Pos	Pos	Pos	Pos	Pos	Mandible	5.5	4	12	F		Neoadjuvant chemo --> surgery, RT, adjuvant chemo --> NED				ANED				21 mo
Le Loarer	31383960	EWSR1-TFCP2	Epithelioid cell RMS		Pos	Pos	Neg	Pos	Pos	Maxilla	6	26	11	F		Chemo --> radiotherapy --> progression (unknown time frame)				DOD				Unknown
Le Loarer	31383960	EWSR1-TFCP2	Epithelioid cell RMS		Neg	Pos	Pos	Neg	Pos	Mandible	3.4	3	25	M		Surgery --> chemo --> NED				ANED				20 mo
Zhong	37545350	FUS-TFCP2	Spindle–epithelioid cell rhabdomyosarcoma		Pos	Pos	Pos	Pos	Pos	Mandible	4.5 × 3.3 × 2.2 cm	Ki-67 30%	26	M		Resection --> recurrence after 2 month --> repeat surgery at 4-month mark --> recurred 2 months later --> died 3 months later				DOD	2 mo			9 mo
Xu	33382123	FUS-TFCP2	Spindle–epithelioid cell rhabdomyosarcoma		Pos	Pos	Pos	Pos	Pos	Mandible			22	M		Unknown				Unknown				
Xu	33382123	FUS-TFCP2	Spindle–epithelioid cell rhabdomyosarcoma		Pos	Pos	Neg		Neg	Mandible			34	M						ANED				10 mo
Koutlas	32504289	EWSR1-TFCP2	Spindle–epithelioid cell rhabdomyosarcoma		Pos	Pos	Neg	Pos	Pos	Mandibular	7 × 1.8 × 1 cm	>10/10	15	M	Fetal hepatoblastoma, mixed epithelial/mesenchymal type at 2 years with lung mets, treated with cisplatin, doxorubicin, 5FU and vincristine --> pulm recurrence 2 years later, resected, irinotecan, vincristine, erlotinib --> remission negative for Li-Fraumeni	Resection with LN dissection --> alternating courses of vincristine, actinomycin D, and cyclophosphamide with etoposide and ifosfamide --> developed met to T7 --> radiation, switched to irinotecan and temozolamide, patient still undergoing treatment at time of paper	Resection with LN dissection			Unknown				Unknown
Panferova	35768243	EWSR1-TFCP2	Spindle cell RMS		Neg		Pos	Pos	Pos	Mandiublar	64mm × 18mm × 44mm	Ki-67 50%	16	F		Resection with negative margins, negative MRI --> originally treated with doxorubicin, cisplatin --> showed local relapse after 1 cycle with mets to LN, C1-C2, iliac wing (tumor progression after 2 months) --> switched to ifosfamide, vincristine, dactinomycin, progressed after 2 cycles (4.5 months after primary diagnosis) --> radiation to C1-C2 + vincristine, irinotecan, temozolamide + crizotinib (250 mg daily) --> palliative 6.5 months after primary diagnosis, death 5 months later			54 Gy total to C1/2	DOD	1 mo	2 mo	1 mo	11.5 mo
Schopf	38168093	FUS-TFCP2	Pleomorphic spindle and epithelioid cell RMS	CDK2NA frameshift del; CCND2 high expression			Pos			Maxillary bone/palate			38	M		Neoadjuvant doxorubicin/ifosfamide, surgery, adjuvant RTX --> relapse at 28 mo --> surgery (5 mo), gemcitabine/docetaxel (2 mo, PD), eribulin (3 mo, MR), crizotinib (3 mo, PD), surgery (3 mo), ifosfamide (2 mo, PD)				DOD	28 mo		N/A	48 mo
Schopf	38168093	EWSR1-TFCP2	Spindle cell RMS	CDK2NA loss			Pos			Mediastinum			60	M		Surgery + XRT --> lung mets at 7 mo --> Doxorubicin/olaratumab (2 mo, PD), trabectedin (2 mo, PD), crizotinib (5 mo, MR)				DOD	N/A		7 mo	20 mo
Schopf	38168093	FUS-TFCP2	Spindle	CDK2NA loss			Pos			Maxilla			48	M		Neoadjuvant vincristine/ifosfamide/actinomycin, surgery, adjuvant vincristine/ifosfamide/actinomycin --> lung, LN, bone mets at 9 mo --> Topotecan/carboplatin/cyclophosphamide/etoposide (4 mo, PD), crizotinib (2 mo, n.e.)				DOD	N/A		9 mo	16 mo
Schopf	38168093	FUS-TFCP2	Epithelioid cell RMS	CDK2NA loss			Pos			Occipital/nuchal soft tissue			35	M		Surgery --> relapse 4 mo --> Surgery (9 mo), surgery, adjuvant RTX (12 mo, n.a.), surgery (4 mo, n.a.), surgery (1 mo), doxorubicin/ifosfamide (3 mo, SD), pazopanib (1 mo, PD), RTX (1 mo, PD), ceritinib (1 mo, n.e.)				DOD	4 mo		13 mo	42 mo
Schopf	38168093	FUS-TFCP2	Spindle–epithelioid cell rhabdomyosarcoma	CDK2NA frameshift del; CCND2 high expression			Pos			Occipital/nuchal soft tissue			40	F		Surgery --> relapse 7 mo --> surgery, adjuvant RTX (16 mo), surgery, adjuvant doxorubicin/ifosfamide (5 mo, n.a.), surgery (6 mo), surgery (2 mo), trabectedin/olaparib (4 mo, SD)				DOD	7 mo		N/A	42 mo
Schopf	38168093	FUS-TFCP2	Unspecified RMS	CCND2 gain and high CCND2 expression			Pos			Iliac bone			17	F		Neoadjuvant vincristine/ifosfamide/doxorubicin/actinomycin/etoposide, neoadjuvant RTX, surgery, maintenance chemotherapy with trofosfamide/idarubicin/etoposide (6 mo) --> relapse with distant mets 14 mo --> Topotecan/cyclophosphamide, Irinotecan/temozolomide (7 mo, SD), trofosfamide/idarubicin/etoposide/pazopanib (2 mo, PD), alectinib (3 mo, PD), RTX (palliative, n.e.), lorlatinib (2 mo, n.e.)				DOD	14 mo		14 mo	34 mo
Schopf	38168093	FUS-TFCP2	Unspecified RMS				Pos			Maxillary bone			14	F		Vincristine/doxorubicin/cyclophosphamide/ifosfamide/etoposide (4 mo, SD) --> progression at 6 mo --> Vincristine/ifosfamide/actinomycin (1.5 mo, PD), carboplatin/etoposide, maintenance chemotherapy with trofosfamide/idarubicin/etoposide, cyclophosphamide/vinblastine (3 mo, SD), RTX (palliative, n.e.)				DOD		6 mo	N/A	14 mo
Schopf	38168093	EWSR1-TFCP2	Unspecified RMS	CDK2NA loss			Pos			Mandible			9	F		Neoadjuvant ifosfamide/vincristine/actinomycin --> progression 2 mo --> surgery, maintenance therapy with crizotinib (9 mo, n.a.), vincristine/irinotecan/temozolomide/olaparib (7 mo, SD), RTX (palliative, n.a.)				DOD	N/A	2 mo		25 mo
Schopf	38168093	FUS-TFCP2	Spindle–epithelioid cell rhabdomyosarcoma	CDK2NA loss; CCND2 high expression			Pos			Temporal/sphenoid bone			15	F		Neoadjuvant ifosfamide/vincristine/actinomycin/doxorubicin, neoadjuvant RTX --> local progression 5 mo --> surgery, temozolomide/irinotecan (2 mo, PD), surgery, gemcitabine/docetaxel (1 mo, PD)				DOD		5 mo		9 mo
Schopf	38168093	FUS-TFCP2	Spindle–epithelioid cell rhabdomyosarcoma	CDKN2A loss; CCND2 gain and high CCND2 expression			Pos			Mandibular head/temporal bone			49	F		Neoadjuvant docetaxel/cisplatin/5-fluorouracil, surgery, adjuvant RTX --> distant met 12 months --> surgery (2 mo), pembrolizumab (2 mo, PD), doxorubicin/ifosfamide and hyperthermia (8 mo, PR), surgery (4 mo), paclitaxel/gemcitabine (3 mo, n.e.)				DOD			12 mo	36 mo
Schopf	38168093	EWSR1-TFCP2	Epithelioid cell RMS	CDK2NA loss			Pos			Shoulder soft tissue			25	F		Surgery, adjuvant RTX --> distant met 6 mo --> surgery (6 mo), surgery (2 mo), doxorubicin/ifosfamide (3 mo, SD), RTX (palliative, n.a.)				LTFU			6 mo	19 mo
Schopf	38168093	EWSR1-TFCP2	Spindle cell RMS	CDK2NA loss			Pos			Ethmoid cells/frontal sinus			58	M		Neoadjuvant doxorubicin/ifosfamide --> local progression 2 months --> “Ifosfamide/vincristine/doxorubicin/actinomycin, RTX, maintenance therapy with cyclophosphamide/vinorelbine (17 mo, PR), RTX (definitive, 5 mo, PD), topotecan/cyclophosphamide (2 mo, PD), crizotinib (1 mo, n.e.)”				DOD		2 mo		33 mo

NA: not applicable.

## Data Availability

The raw data supporting the conclusions of this article may be made available by the authors on reasonable request to the corresponding author.

## Supplementary Materials

The following supporting information can be downloaded at https://www.mdpi.com/article/10.3390/cancers17091441/s1: Figure S1. Outcomes based on fusion partner.

## Abbreviations

IE	Ifosfamide and etoposide
VAC	Vincristine, actinomycin, and cyclophosphamide
VAI	Vincristine, doxorubicin, and ifosfamide
VDC	Vincristine, doxorubicin, and cyclophosphamide
