# Supplementary material for: TFCP2 Fusion-Positive Rhabdomyosarcomas: A Report of 10 Cases and a Review of the Literature
Source: Cancers (Basel). 2025 Apr 25;17(9):1441. doi: 10.3390/cancers17091441 (PMC12070825; doi:10.3390/cancers17091441)
Supplement: Supplementary file 1 [file cancers-17-01441-s001.zip › cancers-3545189-supplementary.pdf]

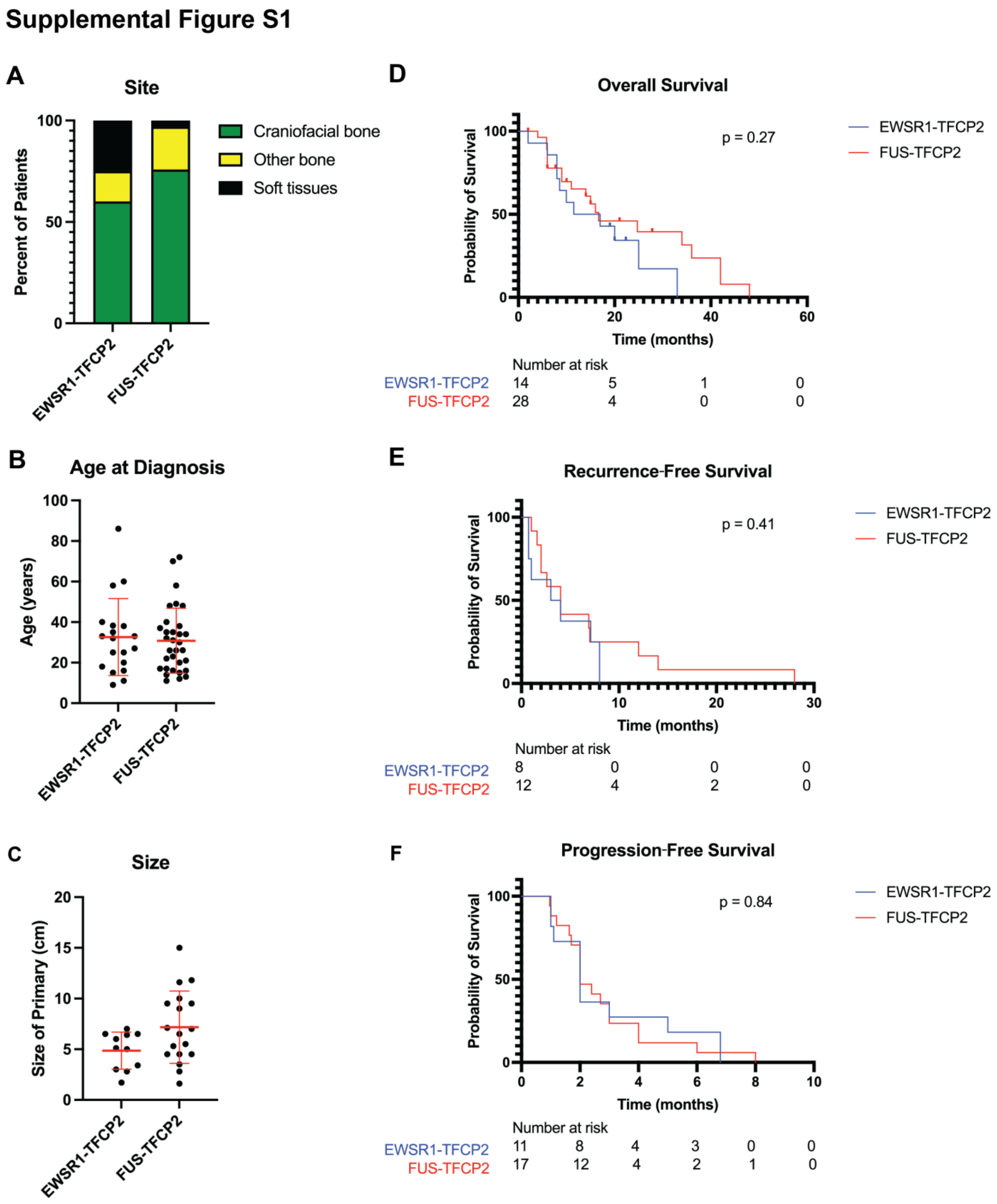

Supplemental Figure S1. Outcomes based on fusion partner.(A) Distribution of primary tumor size by fusion partner. Chi square p value = 0.0496. **(B)** Age at diagnosis, t test p value = 0.7226. Overall survival of the cohort (n = 43). **(C)** Size of primary tumor, t test p value = 0.0562. **(D)** Overall survival, **(E)** Recurrence-free survival, and **(F)** progression-free survival based on fusion partner. Number at risk are shown below the Kaplan Meier curves for reach. P values from log rank tests are displayed on the graphs.
